# Passenger Lymphocyte Syndrome and Autoimmune Hypothyroidism Following Hematopoietic Stem Cell Transplantation

**DOI:** 10.1155/2022/1690489

**Published:** 2022-06-23

**Authors:** Denis F. Noubouossie, Mohammed I. A. Zaanona, Luciano J. Costa, Huy P. Pham, Marisa B. Marques, Antonio Di Stasi

**Affiliations:** ^1^Division of Laboratory Medicine, Department of Pathology, University of Alabama at Birmingham, Birmingham, AL, USA; ^2^Division of Hematology and Oncology, Department of Medicine, O'Neal Comprehensive Cancer Center, University of Alabama at Birmingham, Birmingham, AL, USA; ^3^Be The Match Seattle Collection Center, National Marrow Donor Program, Seattle, WA, USA

## Abstract

We present the case of a 24-year-old male, who received a minor ABO-incompatible allogeneic hematopoietic stem cell transplant (HSCT, blood group O^+^ ⟶ A^+^) from an HLA-matched unrelated female donor, as consolidation therapy for relapsed precursor-B-cell acute lymphoblastic leukemia. The donor had a known history of Hashimoto's thyroiditis before HSCT. At day +10 posttransplant, the patient developed severe hemolysis, which required emergent red blood cell exchange. Additionally, about a year posttransplant, he had circulating antithyroglobulin antibodies, decreased free-T4 (fT4) and increased serum thyroid-stimulating hormone (TSH). The potential causes of the posttransplant hemolytic episode and hypothyroidism are discussed. While the hemolysis was worsened by the transfusion of A red blood cells (RBCs) in the context of passenger lymphocyte syndrome, the thyroid dysfunction might be explained by an autoimmune disease transferred from the donor. The case highlights the possibility of several non-relapse-related complications of HSCT occurring in the same patient. It is critical that such adverse outcomes are distinguished from classical graft-versus-host disease (GVHD) for adequate recipient counseling, posttransplant screening, and prompt treatment.

## 1. Introduction

Allogeneic hematopoietic stem cell transplantation (HSCT) is a curative procedure for selected malignant and non-malignant hematological diseases [1]. Potential complications include relapse of the disease intended to be cured and non-relapse-related toxicities, which altogether contribute to reduced quality of life and significant mortality [2–5]. Non-relapse-related adverse outcomes include toxicity of peritransplant therapies and graft-versus-host disease (GVHD) due to the attack of recipient's tissue by donor effector cells.

Here, we report the case of a patient who received an HSCT from an HLA-matched unrelated donor with a known history of chronic lymphocytic thyroiditis, also known as “Hashimoto's thyroiditis” (HT). In the early posttransplant period, he experienced sudden hemolysis in the context of passenger lymphocyte syndrome (PLS) and symptomatic hypothyroidism a year after transplant.

The potential mechanisms that led to acute hemolysis and autoimmune hypothyroidism are discussed in order to highlight the importance of assessing transplant donors for autoimmune conditions. Proper patient counseling, posttransplant screening, and timely treatment of these complications are essential to long-term success of HSCTs.

## 2. Case Presentation

The patient was a 24-year-old white male without pretransplant autoimmune conditions, diagnosed with precursor-B-cell acute lymphoblastic leukemia (ALL) in 2008. Cytogenetic analysis demonstrated near hypertriploid karyotype with trisomy 4, 10, and 17. After being treated with multiagent chemotherapy according to the AALL0232 protocol, he achieved complete remission. Six years later, he relapsed and was treated with the pediatric R3 protocol, achieving a second complete morphologic remission. He then received consolidation with cyclophosphamide 70 mg/m^2^ and total body irradiation (TBI, 1000 cGy) followed by an allogeneic HSCT from an HLA-matched unrelated female donor in February of 2015. Prophylaxis of GVHD consisted of a single dose of posttransplant cyclophosphamide (50 mg/Kg), tacrolimus, and mycophenolate mofetil. Donor and Recipient HLA type was A^*∗*^03 : 01/24 : 02, B^*∗*^07 : 02/50 : 01, C^*∗*^07 : 02/06 : 02, Bw6, DRB1^*∗*^04 : 01/07 : 01, and DQB1^*∗*^03 : 01/02 : 02. The dose of infused total nucleated cells was 1.18 × 10^9^ cells/kg with 92% viability, including 1.11 × 10^7^ CD34^+^ cells/kg and 2.44 × 10^8^ CD3^+^ cells/kg of body weight. Full donor CD3, CD20, and CD15 chimerism was attained by day +28. Of note, this was a minor ABO-incompatible HSCT as the patient's blood group was A^+^, whereas the donor was O^+^.

**Figure 1:**
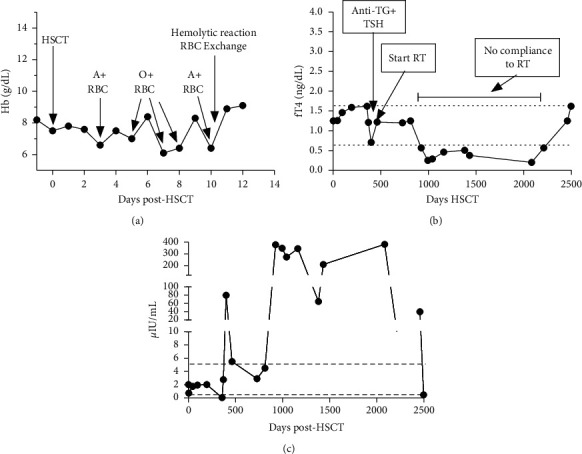
Onset of severe immune hemolysis and autoimmune thyroiditis in a HSCT recipient. (a): Hemoglobin level and timeline of red blood cell (RBC) transfusions up to day +12 after HSCT. Note that a severe hemolytic transfusion reaction occurred after transfusion of group A iRBCs on day +10, which prompted an emergent red blood cell exchange. (b): Free-T4 (fT4) and (c) thyroid-stimulating hormone (TSH) levels up to day +2500 post-HSCT. Note the appearance of antithyroglobulin (Anti-TG) in the serum followed by the peak of TSH and the drop of fT4, indicating autoimmune thyroiditis.

On day +3 post-HSCT, the patient developed a nonpruritic, erythematous rash on his chest, abdomen, and extremities. A skin biopsy revealed interface dermatitis consistent with a drug reaction. He was transfused one unit of irradiated iRBCs on each of day +3, +5, +7, +8, and +10 (Figure 1(a)). The blood type of the iRBC units was A, O, O, O, and A, respectively. Additionally, two units of irradiated apheresis platelets (iPLTs) were transfused on days +3 and +10. Shortly after receiving the iRBC unit on day +10, the patient complained of worsening back pain and was noted to have decreased urine output, which prompted a transfusion reaction workup (Table 1). The blood samples collected before and after the transfusion were reported as A^+^ with mixed-field reaction, consistent with the presence of group O RBCs likely from his recent transfusions of O iRBCs. Although the plasma color posttransfusion was amber and did not show overt hemolysis, the serum haptoglobin was low and lactate dehydrogenase (LDH) and bilirubin levels were higher than pretransfusion values. A posttransfusion direct agglutination test (DAT) was also microscopically positive for IgG and C_3_ (1^+^) compared with a DAT on his pretransfusion blood sample, which was negative for IgG and microscopically positive for C_3_. His antibody screen was negative both pre- and posttransfusion, indicating that a non-ABO circulating alloantibody was unlikely present. At this point, it was evident that the blood bank had erroneously issued a group A iRBC unit using the electronic crossmatching method only. A serologic posttransfusion crossmatch could not be performed because the iRBC unit was not returned to the laboratory. However, clerical checks confirmed that the most recently given iRBC unit was A^+^.

During the transfusion reaction workup, anti-A was newly identified in the patient's plasma at a titer 8, which was present in both samples collected immediately before and following the transfusion implicated in the reaction. A posttransfusion urinalysis showed moderate (3+) hemoglobinuria and 3–10 red blood cells/40x field, and both of which were absent in a urine sample collected before the transfusion of the triggering iRBC unit on day +10. Blood urea and creatinine both rose from normal values pretransfusion to abnormally high values postreaction (Table 1). The workup collectively indicated an acute hemolytic transfusion reaction with acute kidney injury, likely due to anti-A from the donor reacting with transfused and patient's own group A RBCs. In addition to receiving corticosteroids, an emergent red blood cell exchange was performed. During the procedure, approximately 70% of the patient's circulating red cells were replaced with seven units of group O iRBCs to achieve a final hematocrit of 24%. Following the exchange, the patient's clinical condition improved gradually, including his kidney function and hemoglobin level, which rose and remained above 7 g/dL. The haptoglobin returned to normal within 48 hours, and his blood type converted to group O by day +94.

Around days +60 and +111, the patient developed acute gastrointestinal and cutaneous GVHD, respectively. He received systemic corticosteroids, immunosuppression with tacrolimus, and later, rituximab. As a steroid-sparing agent and for poor compliance with tacrolimus, he was also started on mycophenolate mofetil.

In light of the history of HT in the donor, the patient was monitored with serum free-T4 (fT4) levels (Figure 1(b)), thyroid-stimulating hormone (TSH) (Figure 1(c)) and the presence of autoantibodies directed against the thyroid. His thyroid panel pretransplant was normal, including the absence of detectable antibodies. On transplant day +3, he also had normal TSH and fT4 of 0.744 *µ*IU/mL (reference range: 0.45–5.5 *µ*IU/mL) and 1.25 ng/dL (reference range: 0.62–1.57 ng/dL), respectively. On re-evaluation on day +46, TSH and T4 levels remained within the normal range while antithyroglobulin (anti-TG) and antithyroid peroxidase (anti-TPO) were undetectable. However, on day +358, TSH was 0.047 *µ*IU/ml and fT4 was 1.62 ng/dL suggesting an initial hyperthyroid phase followed by a hypothyroid phase from day +372 with a high TSH (79.6 *µ*IU/mL). Anti-TG was detected at a titer of 3 U (reference range: <0.5 U) at this time indicating the development of autoimmune hypothyroidism. Concurrent decrease thyroid function and the appearance of anti-TG antibodies in the serum suggest that complement-mediated injury may have also contributed the thyroid gland injury. Therefore, thyroid replacement therapy was initiated. An ultrasound on day +511 revealed a normal thyroid gland without evidence of nodules or parenchymal abnormalities and no increased blood flow. Grade 2 parenchymal echogenicity, high vascularity, and paratracheal lymph nodes enlargement typically seen on thyroid ultrasound during hypothyroidism were missing possibly because the patient has already been on replacement therapy for several months and was in an euthyroid state.

Further follow-up on day +813 showed normal thyroid function and undetectable antithyroid antibodies. However, the patient was not compliant with his thyroid hormone replacement therapy for a long period during which fT4 level was low and TSH was high (Figures 1(b) and 1(c)). During this period, the patient complained of fatigue and had two admissions to the hospital for sepsis for which he recovered. Following several sessions of patient education on the importance of taking prescribed medications, the patient resumed thyroid hormone replacement therapy and both fT4 and TSH levels returned to normal values around day +2498 posttransplant. Seven years later, the patient remains on thyroid replacement therapy. The latest posttransplant course was complicated by oral and cutaneous chronic GVHD.

## 3. Discussion

This patient experienced immune hemolysis requiring red blood cell exchange on day +10 and developed autoimmune hypothyroidism approximately a year after receiving a minor ABO-incompatible allogeneic HSCT. To our knowledge, this combination of immunologic complications has not been reported in the literature to date. Instead, there are case reports of patients who developed one [6–10] or the other [11–14] following HSCT.

Several mechanisms can potentially explain the occurrence of hemolysis in minor ABO-incompatible HSCT. Isoagglutinins (anti-A in this case) contained in the graft and directed against the recipient's ABO antigens can be passively transferred during transplant, resulting in immediate hemolysis. This is more likely to occur if the donor has high titer isoagglutinins, the plasma volume in the HSCT product is large, and the recipient has a small body weight. However, it is unlikely that passive transfer of anti-A was responsible for the hemolytic reaction observed in this patient since laboratory markers of hemolysis were not present until day +10 before the transfusion of a group A iRBC unit. Of note, the patient was still typing as blood group A in a blood sample collected on day +10 prior to the triggering transfusion event, indicating the presence of a significant number of recipient-type red cells before the transfusion. In addition to detecting anti-A in his plasma, his DAT was also positive on the same blood sample, suggesting in vivo binding of the antibody to his red cells before the transfusion. Thus, one can reasonably speculate that transfusion of the wrong blood type exacerbated an already ongoing, subclinical, or imminent hemolytic process.

Indeed, this patient should not have received a group A iRBCs. Although the standard operating procedure in our institution at the time of this event allowed the release of RBC units based on the patient's historic blood type when the antibody screen was negative, an exception to this rule should have been applied for allogeneic HSCT recipients. When the error occurred, the blood bank staff had to manually check the patient's status and the type of ABO incompatibility, and issue compatible iRBC units according to a table [15]. If this step had occurred, the patient would have received group O iRBCs. Since this event, an electronic process to ensure that the appropriate blood type of RBCs is released to HSCT recipients every time has replaced the manual check.

Passenger lymphocyte syndrome (PLS) is the most likely underlying condition that predisposed the patient to the severe hemolysis observed following the transfusion of A iRBCs. PLS occurs when viable B-lymphocytes in the graft produce incompatible antibodies directed against the recipient's red cell antigens. In this case, B-lymphocytes from the donor produced anti-A (and anti-B) which reacted with the A iRBCs transfused. Hemolysis associated with PLS occurs usually five to 15 days posttransplant and rarely after eight weeks [15–17]. While clinically significant hemolysis occurs in only 10–15% of minor ABO-incompatible HSCT [16], severe and fatal hemolysis has been reported [7]. PLS risk factors include transplant from unrelated or non-HLA-matched sibling donors, recipient of blood group A, HSC apheresis product, and usage of calcineurin inhibitors without methotrexate as immunosuppressive regimen posttransplant [15, 18]. Some of these risk factors were present in our patient.

Besides requirements for blood products, no guidelines exist to prevent hemolytic reaction following HSCT in minor ABO incompatibility. However, reduction of plasma volume of the graft is performed in some institutions to minimize the transfer of isoagglutinins to the recipient [15, 19]. Other authors have performed pretransplant red blood cell exchange to replace recipient's red cells with group O RBCs. However, hyperhemolysis involving donor's O RBCs may occur due to adsorption of antigen-expressing microvesicles from the recipient's red cells [18]. Neither graft plasma reduction nor pre-HSCT red blood cell exchange was performed in this patient. Instead, the patient had a posthemolysis exchange with group O iRBCs, to which he responded well.

About a year after HSCT, the patient developed a symptomatic hypothyroidism and has been dependent on thyroid hormone replacement therapy. The presence of symptoms with laboratory evidence of decreased thyroid function concurrently with the appearance of anti-TG antibodies in the serum suggests that the thyroid disease is autoimmune. Although more specific antithyroxin peroxidase antibodies were below detection level at Day +46 posttransplant, the presence of HT in donor raises the possibility of transferred HT to the patient. Similarly to other examples of HT post-HSCT previously reported [11–14], the disease was present in the donor before HSC collection but absent in the recipient before HSCT. This latency time is consistent within the range of 12 to 48 months previously described. Both overt and subclinical hypothyroidism have been reported, and most patients received thyroid hormone replacement. Several reported cases experienced acute GVHD, and a few developed chronic GVHD. TBI was part of the conditioning regimen in several cases.

The pathogenesis of transferred autoimmune thyroiditis in the context of HSCT is not well-understood. A large cohort study from China suggested that the most significant triggering event was the transfer of donor lymphocytes and their effect on the recipient's thyroid [20]. Other significant risk factors identified in the cohort study from China included the use of a female donor, HSCT for chronic myeloid leukemia, the HLA B46, DR9 loci, and the A2B46DR9 haplotype [20]. Indeed, there is a strong genetic susceptibility associated with HT [21]. Polymorphisms in major immune-regulatory genes including HLA, cytotoxic T-lymphocyte antigen-4, and Protein Tyrosine Phosphatase Nonreceptor-Type 22 gene have also been linked to HT [21]. Pretransplant HLA showed that the donor and the patient had the DRB1^*∗*^04-DQB1^*∗*^0301 haplotype which is reportedly associated with HT in Caucasians [22]. Other predisposing HLA alleles not present in this patient have also been associated with HT, including DR3, DR5, DQ7, and DQw7 [21]. Since gene polymorphisms can modulate mature immune cell functions, it is speculated that HT's susceptibility could be transferred from the donor to the recipient through the engrafted HSCs. Supporting this hypothesis, the transmission of HT has been reported following the transplant of selected CD34^+^ peripheral blood stem cells [14]. Other potential contributing factors to thyroid dysfunction post-HSCT include the effect of conditioning chemotherapy, radiation damage to the thyroid before transplantation, graft-versus-host disease, and the general immune dysregulation associated with the transplant [12].

In summary, HSCT can potentially transfer humoral and cellular immune effectors and susceptibility genes for autoimmune diseases from the donor to the recipient. While minor ABO incompatibility and the diagnosis of HT do not preclude donor suitability for donation, they increase the risk of non-relapse-related complications of HSCT. They must be recognized for donors' evaluation [23], recipient counseling, posttransplant screening, prompt treatment, and differential diagnosis from classic GVHD manifestations and other toxicities, requiring specific therapeutic measures. Some of such manifestations can be considered under the GVHD spectrum.

## Figures and Tables

**Table 1:** Laboratory parameters before and after the transfusion reaction.

Parameters	Before transfusion	After transfusion
Antibody screening	Negative	Negative
ABO forward type	A (mixed-field reaction)	A (mixed-field reaction)
Crossmatch	Compatible (electronic)	Incompatible
Urine hemoglobin	Negative	3+ (moderate)
Urine RBCs @ 40x	Negative	3–10/Field
BUN (Ref:5–22 mg/dL)	7 mg/dL	30 mg/dL
Creatinine (ref: 0.4–1.2 mg/dL)	0.5 mg/dL	1.6 mg/dL
Haptoglobin (ref: 33–271 mg/dL)	Not done	3 mg/dL
LDH (ref: 120–240 IU/L)	Not done	858 IU/L
Indirect bilirubin	0.5 mg/dL	7.5 mg/dL

RBC: red blood cell; BUN: blood urea nitrogen; LDH: lactate dehydrogenase. Ref: reference range; IU: international unit.

## Data Availability

The laboratory results supporting our conclusion were obtained from the Electronic Medical Record System of UAB, with patient's informed consent.
